# A leucine-rich repeat-receptor-like kinase gene *SbER2–1* from sorghum (*Sorghum bicolor* L.) confers drought tolerance in maize

**DOI:** 10.1186/s12864-019-6143-x

**Published:** 2019-10-15

**Authors:** Hanshuai Li, Xiaodong Han, Xinxiang Liu, Miaoyi Zhou, Wen Ren, Bingbing Zhao, Chuanli Ju, Ya Liu, Jiuran Zhao

**Affiliations:** 1 Beijing Key Laboratory of Maize DNA Fingerprinting and Molecular Breeding, Maize Research Center, Beijing Academy of Agriculture & Forestry Sciences, Beijing, 100097 People’s Republic of China; 2 College of Life Sciences, Capital Normal University, Beijing, 100048 People’s Republic of China

**Keywords:** Drought, Maize, *SbER2–1*, Water-use efficiency, Lignin accumulation

## Abstract

**Background:**

*ERECTA* (*ER*) is a leucine-rich repeat-receptor-like kinase gene (LRR-RLK) encoding a protein isolated from *Arabidopsis*. Although the regulatory functions of *ER* genes have been widely explored in plant development and disease resistance, their roles in drought stress responses remain to be clarified.

**Results:**

In this study, we cloned and characterized two *ER* genes, *SbER1–1* and *SbER2–1*, from the drought-tolerant model plant sorghum (*Sorghum bicolor* L.). Under drought stress, the two genes were expressed in the leaves and stems but not in the roots, and *SbER2–1* transcript accumulation in the stem was increased. *SbER2–1* was localized both on the plasma membrane and in the chloroplast. Moreover, *SbER2–1* expression in *Arabidopsis* and maize conferred increased drought tolerance, especially in regard to water-use efficiency, increasing the net photosynthetic rate in maize under drought stress. Based on RNA-Seq analysis together with the physiological data, we conclude that the transgenic maize plants have upregulated phenylpropanoid metabolism and increased lignin accumulation under drought stress.

**Conclusions:**

Our results demonstrate that *SbER2–1* plays an important role in response to drought stress. Furthermore, photosynthetic systems and phenylpropanoid metabolism are implicated in *SbER2–1*-mediated drought stress tolerance mechanisms. The use of genetic engineering to regulate *SbER2–1* expression in plants and to breed new varieties tolerant to drought is a research field full of potential.

## Background

Maize crops account for the largest planting area and highest yield of any crop in China, where many growing areas are concentrated in arid and rainless regions [1]. Drought is becoming an important limiting factor in maize production [2]. Water shortages are particularly serious in the northern regions of China, where frequent droughts have caused significant losses in maize production [3]. In recent years the mean annual agricultural disaster area in Northeast China is approximately 6.43 × 10^4^ km^2^, including 3.9 × 10^4^ km^2^ (60.5%) caused by drought disaster [4]. Thus, the identification of drought-tolerance and water-saving genes using modern biotechnology is urgently required to develop and cultivate new drought-tolerant varieties that will reduce the production losses caused by drought stress.

Receptor-like protein kinases (RLKs), which represent the largest gene family in plants, play critical roles in plant developmental processes, signaling networks, and stress resistance [5]. In recent years, there has been increased research on the role of RLKs in drought stress responses, focusing mainly on leucine-rich repeat-type RLKs (LRR-RLKs), receptor-like cytoplasmic kinases (RLCKs), and S-domain-type RLKs (S-RLKs). LRR-RLKs are a subpopulation of signaling receptors that are ubiquitous in plants and regulate a variety of signaling pathways [6]. A rice LRR-RLK gene, *FON1*, is induced by drought and ABA treatment, and *FON1* overexpression in transgenic rice plants increases drought tolerance and sensitivity to ABA compared with that in plants with *FON1* silenced through RNA interference [7]. Overexpression of the LRR-RLK gene *OsLRK2* increases drought tolerance and tiller number in rice via increased branch development [8]. Guo et al. (2016) isolated an alfalfa LRR-RLK gene, *MsSIK1*, which was identified as a water-use efficiency (WUE)-related gene due to its strong expression in response to dehydration [9]. Overexpression of an *Arabidopsis* cysteine-rich receptor-like protein kinase, *CRK5*, enhances abscisic acid sensitivity and confers drought tolerance [10]. The rice S-domain RLK gene *OsSIK2* enhances tolerance to drought stress by activating expression of POX-1, POX-2, and POD or through detoxification of reactive oxygen species (ROS) [11].

*ERECTA* (*ER*) is an *Arabidopsis* LRR-RLK gene. In *Arabidopsis*, *ER* genes function in physiological and biochemical processes such as coordination of photosynthesis and transpiration efficiency, regulation of the growth and development of aboveground organs (such as flowers and leaves), hormone and light regulation, disease resistance signal recognition, and transduction [12, 13]. The *Arabidopsis ER* genes improve transpiration efficiency by influencing the development of epidermal cells and mesophyll cells, stomatal density, and leaf porosity. In addition, *ER* balances transpiration and photosynthesis by improving leaf characteristics and the photosynthetic capacity of mesophyll cells [14]. Overexpression of the *PdER* gene from poplar in *Arabidopsis* results in increased root length and leaf area at the seedling stage, and greatly enhances long-term WUE [15]. Liu et al. (2012) demonstrated significantly higher expression of the rice *ER* gene in phyB mutants compared with the wild type. In addition, a phyB mutant upregulates *ER* gene expression to regulate leaf stomatal density and the plant transpiration rate [16]. Overexpression of *AtER* in *Arabidopsis*, rice, and tomato enhances the heat tolerance and biomass of these transgenic crops [17]. Du et al. (2018) also reported that auxin and gibberellins are required for ER regulated hypocotyl elongation in shade avoidance in *Arabidopsis* [18].

Although the regulatory functions of *ER* genes in plant development and disease resistance have been widely explored, their roles in the drought stress response remain to be clarified. Sorghum (*Sorghum bicolor* L.) is a well-studied crop with strong drought tolerance that is used as a model plant for drought tolerance, not only to improve its own characteristics but also as a potential source of stress tolerance traits to improve other crops. In the present study, we cloned and characterized *SbER1–1* and *SbER2–1* from the sorghum variety JZ12, which has strong drought tolerance, and investigated the potential of *SbER2–1* overexpression as a means to improve drought tolerance in *Arabidopsis* and maize. We also explored the putative mechanisms underlying the response of *SbER2–1*-transgenic maize to drought stress, with the aim of improving crop growth under water shortage conditions.

## Results

### Genomic sequence characterization of *SbER*

**Fig. 1:**
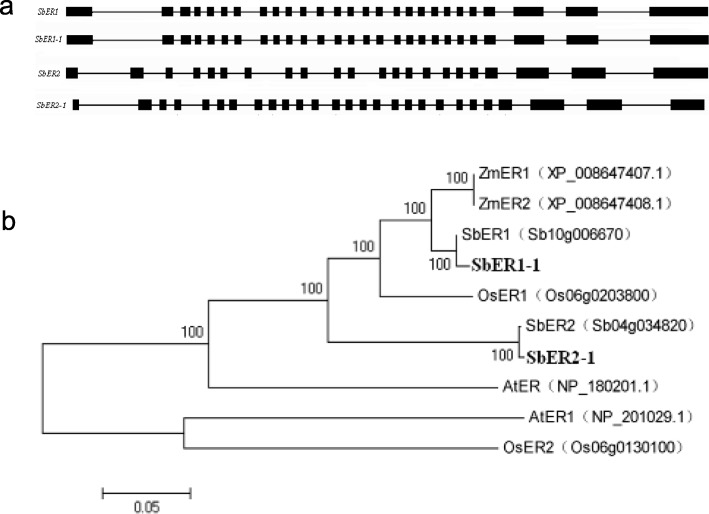
Sequence characteristics and phylogenetic analyses of sorghum *ER* family genes. **a** The exon-intron structure of sorghum *ER* family genes. **b** Phylogenetic tree of *ER* family genes in Arabidopsis (At), maize (Zm), rice (Os), and sorghum (Sb)

Two sorghum *ER* gene family members, Sb10g006670 (*SbER1*) and Sb04g034820 (*SbER2*), were predicted based on BLAST analysis of the cDNA sequences of *ER* genes of *Arabidopsis*, rice, and maize (NM_128190.2, NM_125617.2, NM_001063622.1, NM_001063222.1, XM_008649185.1, XM_008649186.1) against the sorghum GenBank genomic database. Based on the NCBI database, the genomic DNA sequences of *SbER1* (NC_012879.1) and *SbER2* (NC_012873.1) were located on chromosomes 10 and 4, respectively. We obtained the DNA and cDNA sequences of *SbER1–1* and *SbER2–1* from sorghum variety JZ12 by PCR amplification using the designed long fragment-specific amplification primers (Table S3). The *SbER1–1* gene had 26 introns, which is identical to the exon–intron structure of *SbER1*. The exon–intron structure of *SbER2–1* also had 26 introns, while *SbER2* has only 21 introns (Fig. 1a). We further analyzed the encoded amino acid sequences using online software (InterProScan 5, SMART, SignalP v3.0 and TMHMM v2.0). The protein sequences of the sorghum ER family members had a signal peptide region, a LRR region, a transmembrane domain, and a protein kinase domain. The LRR and protein kinase domains exhibited higher sequence conservation than the transmembrane domain, the N-terminal signal sequence, and the C-terminal sequence.

We then carried out sequence similarity analysis by aligning the amino acid sequences encoded by *SbER1–1* and *SbER2–1* with the amino acid sequences of the *Arabidopsis*, rice, and maize ER family members (Additional file 1: Figure S1) [11]. The amino acid sequence identity of *SbER1–1* and *SbER1* was 99.3%, and the amino acid sequence identity of *SbER2–1* and *SbER2* was 97.3% (Additional file 2: Figure S2). Construction of a phylogenetic tree using the neighbor-joining method revealed that the sorghum ER protein and other reported ER proteins belong to the LRR-RLK family (Fig. 1b). Additionally, we analyzed the DNA and amino acid sequence identities of the six sorghum varieties. The identity of the full-length DNA sequences of the *SbER1* and *SbER2* genes of the six sorghum varieties was over 98.0%, and the identities of the cDNA sequences of *SbER1* and *SbER2* from the six sorghum varieties were 99.6 and 96.0%, respectively. Compared to the amino acid sequence of the variety Tx623, we detected few changes in the corresponding amino acid sequences encoded by *SbER1* in the six sorghum varieties. By contrast, the full-length cDNA sequences of *SbER2* in all six varieties had more than 96.0% identity, with more base changes and corresponding amino acid sequence changes (Table 1).

**Table 1:** Analysis of amino acid sequences encoded by *SbER2* in six sorghum varieties

cDNA sequence position (bp)	1383–2	V4A	10028D	Tx623B	Jingliangwu	363C/2691
534	G/A(−)					
763	G/T(−)					
1238		T/G(I/S)	T/G(I/S)			
1451			+C(+P)			
2235			T/G(F/S)			
2236			T/G(F/S)		T/G(I/S)	
2628						A/G(K/E)
2811						A/G(S/G)

Note: Changes to the corresponding coding amino acid sequences are given in parentheses. I, Ile (isoleucine); S, Ser (serine); P, Pro (proline); F, Phe (phenylalanine); K, Lys (lysine); E, Glu (glutamic acid); G, Gly (glycine)

### Analysis of *SbER* expression in response to drought stress

**Fig. 2:**
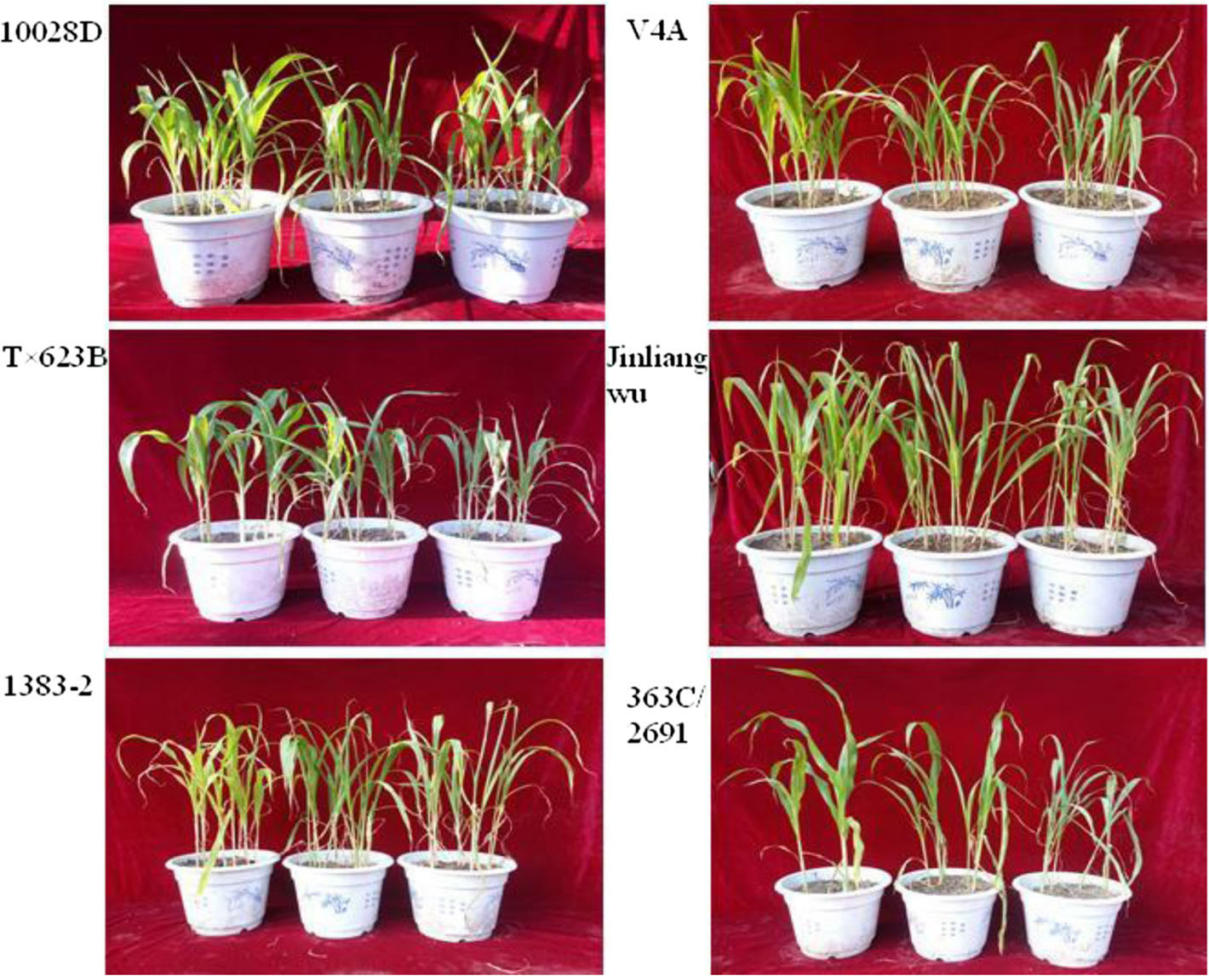
Seedling phenotypes of six sorghum varieties under drought stress. Each variety was subjected to three conditions: from left to right in each photograph, well-watered (WW), moderate drought stress (MS), and severe drought stress (SS)

Six different sorghum varieties were cultivated under well-watered (WW) and drought (moderate stress, MS, and severe stress, SS) conditions (Fig. 2). There were significant differences in the plant water content and fresh biomass of sorghum varieties cultivated under the different conditions (*P* < 0.05), indicating that water stress treatments elicited differential phenotypes in the six varieties (Table 2). In comparison with Tx623B and 363C/2691, Jinliangwu, 10028D, and 1383–2 maintained an increased growth potential under drought stress conditions and showed stronger drought tolerance.

**Table 2:** Leaf water content and biomass of six sorghum varieties under different drought conditions

Variety	leaf RWC(%) WW	leaf RWC(%) MS	leaf RWC(%) SS	Fresh biomass (g) WW	Fresh biomass (g) MS	Fresh biomass (g) SS
10028D	14.63 ± 0.86	9.65 ± 0.07	6.67 ± 0.50	5.31 ± 0.27	3.89 ± 0.57	2.77 ± 0.30
V4A	15.57 ± 0.35	9.67 ± 0.42	5.20 ± 0.57	4.76 ± 0.19	3.26 ± 0.39	2.75 ± 0.03
Tx623B	14.45 ± 0.07	8.80 ± 0.52	6.00 ± 0.42	8.07 ± 0.46	5.51 ± 0.43	2.81 ± 0.51
Jingliangwu	14.97 ± 0.55	8.33 ± 0.42	4.90 ± 0.62	5.78 ± 0.76	4.99 ± 0.18	2.78 ± 0.46
1383–2	17.55 ± 0.64	8.13 ± 0.12	5.40 ± 0.26	5.75 ± 0.08	4.55 ± 0.15	3.69 ± 0.35
363C/2691	14.70 ± 0.10	10.35 ± 0.49	5.20 ± 0.46	6.63 ± 0.18	3.98 ± 0.67	3.48 ± 0.14

Note: Plants were subjected to water stress to maintain a stable absolute soil water content that fluctuated within the ranges of 17–18%, 9–10%, and 6–7% for well-watered (WW) and two drought conditions (moderate stress, MS; severe stress, SS), respectively

**Fig. 3:**
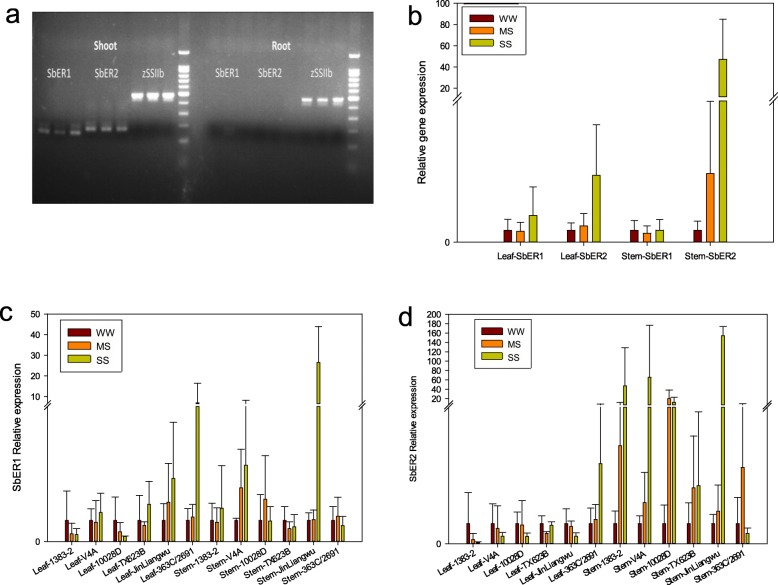
Expression analyses of *SbER* genes. **a** Expression of *SbER* genes in the shoots and roots. zSSIIb is an endogenous reference gene in maize. **b** Expression of *SbER1*and *SbER2* in well-watered (WW) and drought-treated (MS and SS) seedlings of sorghum. **c** Percentage of up- and downregulated *SbER1* genes in sorghum seedlings under the same three conditions. **d** Percentage of up- and downregulated *SbER2* gene in sorghum seedlings under the same three conditions

Quantitative reverse transcription polymerase chain reaction (qRT-PCR) analysis of the stems and roots of sorghum variety 10028D exposed to drought stress showed that *SbER1* and *SbER2* were not expressed in the roots but were expressed in the shoots (Fig. 3a), including both the leaves and the stems (Fig. 3b). In the leaves, there were no significant changes in the *SbER1* expression levels of any of the six sorghum varieties when exposed to MS conditions. Under SS conditions, however, *SbER1* expression was upregulated in sorghum varieties 363C/2691 and Jinliangwu, although not in the other four varieties. In the stem, *SbER1* expression was upregulated only in V4A and 10028D under MS conditions, and only in V4A and Jinliangwu under SS conditions (Fig. 3c). In the leaves, the six sorghum varieties exhibited only limited variation in *SbER2* expression levels under drought conditions, although *SbER2* expression was upregulated in 363C/2691 under SS conditions. In the stem, *SbER2* expression was significantly upregulated in all six varieties under MS conditions. Under SS conditions, *SbER2* expression was significantly upregulated in five of the six sorghum varieties, with 363C/2691 being the exception (Fig. 3d).

In the stem, *SbER2* expression was upregulated to a greater extent than that of *SbER1* in response to drought stress. Furthermore, *SbER2* expression was upregulated to a greater extent in the three most drought-tolerant varieties, Jinliangwu, 10028D, and 1383–2, than in the drought-sensitive Tx623B and 363C/2691. Therefore, we selected *SbER2* for further investigation of genes related to drought stress.

### Subcellular localization of SbER2–1

**Fig. 4:**
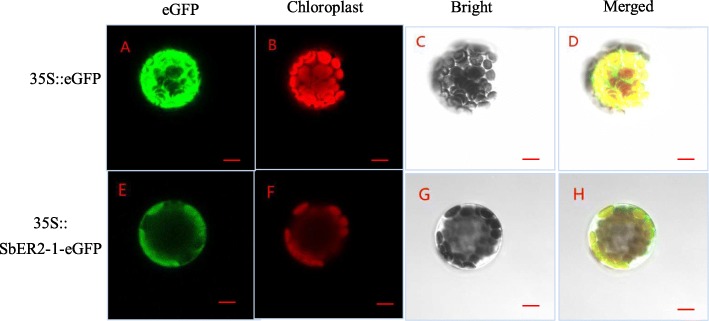
Subcellular localization of SbER2–1 in *Arabidopsis*. Mesophyll protoplasts from *Arabidopsis* transfected with *35S::eGFP* or *35S::SbER2–1-eGFP* were examined by confocal fluorescence microscopy. Scale bars = 5 μm. Representative images are shown

To elucidate the role of SbER2–1 proteins at the cellular level, we investigated its subcellular localization of through transient expression in *Arabidopsis* protoplasts. The *SbER2–1* open reading frame (ORF) (with no stop codon) was fused to the N-terminus of the enhanced green fluorescent protein gene (*eGFP*) in the pE3449 vector with expression driven by the CaMV 35S promoter, generating the *35S::SbER2–1-eGFP* fusion construct. Confocal microscopy revealed fluorescence from the SbER2–1-eGFP fusion protein on the cell membrane and chloroplasts of *Arabidopsis* protoplasts, while eGFP (expressed from the control construct *35S::eGFP*) was dispersed throughout the whole cell (Fig. 4). These observations indicated that the SbER2–1-eGFP fusion protein was localized on the cell membrane and chloroplast, which was inconsistent with the location in the plasma membrane predicted using CELLO v2.5 software. That SbER2–1 was localized not only in the plasma membrane, as predicted, but also in the chloroplast suggested that it plays a vital role in plant photosynthesis.

### Overexpression of *SbER2–1* enhances drought tolerance in *Arabidopsis* and maize

**Fig. 5:**
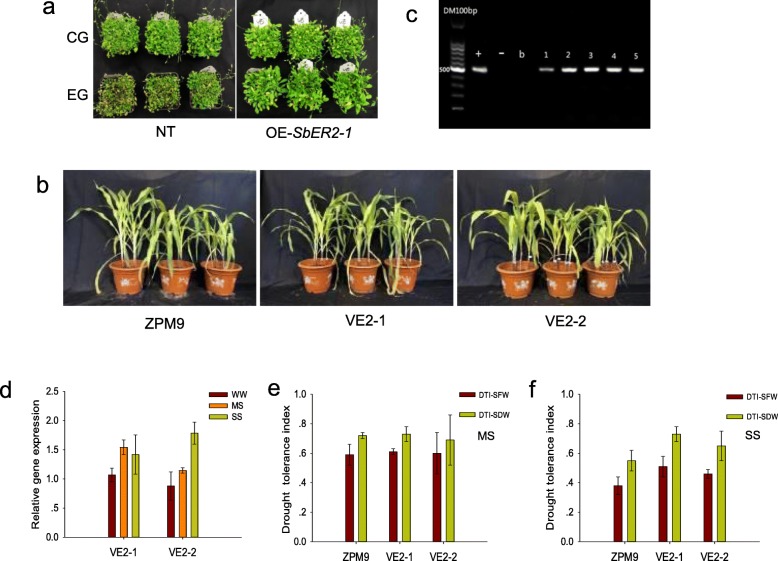
Phenotypes, molecular identification and *SbER2–1* expression level in transgenic plants. **a** Phenotype identification of T_3_ Arabidopsis plant transformed with *SbER2–1*. NT, non-transgenic (wild type); OE-*SbER2–1*, transgenic plants; CG, control group with normal irrigation; EG, experimental group treated with PEG. **b** Plant aboveground phenotypes of transgenic maize lines VE2–1 and VE2–2, as well as NT ZPM9 (CK), under WW (left), MS (middle), and SS (right) conditions. **c** PCR detection of transgenic lines by agarose gel electrophoresis. Transgenic plants (1–5 lane) and positive control (+) show amplified 506-bp fragment; negative control (−) and blank control (**b**) had no amplified fragment. **d** *SbER2–1* expression level of transgenic maize plants under WW, MS, and SS conditions. **e, f** Drought-tolerance index (DTI) of shoot biomass of VE2–1, VE2–2, and ZPM9 maize under MS (**e**) and SS (**f**) conditions, respectively

To further characterize the function of *SbER2–1* under drought conditions, we obtained five *Arabidopsis* and three maize transgenic lines (T_1_). A qRT-PCR analysis indicated that *SbER2–1* was expressed in all of the transgenic plants but not in the non-transgenic (NT) lines. Therefore, we selected three *Arabidopsis* and two maize transgenic lines with higher *SbER2–1* expression for subsequent experiments. T_3_ homozygous lines generated from the selected transgenic lines were used in the following experiments. A phenotypic analysis of drought tolerance of the transgenic *Arabidopsis* lines overexpressing *SbER2–1*, after 2 weeks indicated that all of the leaves of the NT plants were heavily curled, while only a few of the *SbER2–1*-overexpressing plants were affected by the drought stress, with most leaves still green and fully expanded (Fig. 5a).

Next, we investigated drought tolerance in the two transgenic (VE2–1 and VE2–2) and the NT ZPM9 maize lines after exposure to different drought stress conditions for 2 weeks (Fig. 5b). The transgenic lines overexpressed *SbER2–1* by 1.5- to 2.0-fold under MS and SS conditions, respectively, as compared to WW conditions (Fig. 5d). We also analyzed shoot fresh weight (SFW) and shoot dry weight (SDW) using the drought-tolerance index (DTI), which was determined by calculating the ratio of drought to WW treatment values (Fig. 5e,f). Under MS conditions, there were no significant differences in the DTI of SFW and SDW among the ZPM9, VE2–1, and VE2–2 lines. Under SS conditions, the DTIs of the SFW and SDW of VE2–1 and VE2–2 transgenic seedlings were significantly higher than those of the NT ZPM9 lines (*P* < 0.01), which indicated that the transgenic seedlings had greater drought tolerance than the NT ZPM9 seedlings under severe drought conditions. These results suggested that *SbER2–1* overexpression helps maintain plant growth under drought conditions.

### Transcriptome analysis of *SbER2–1* transgenic maize under drought stress

**Fig. 6:**
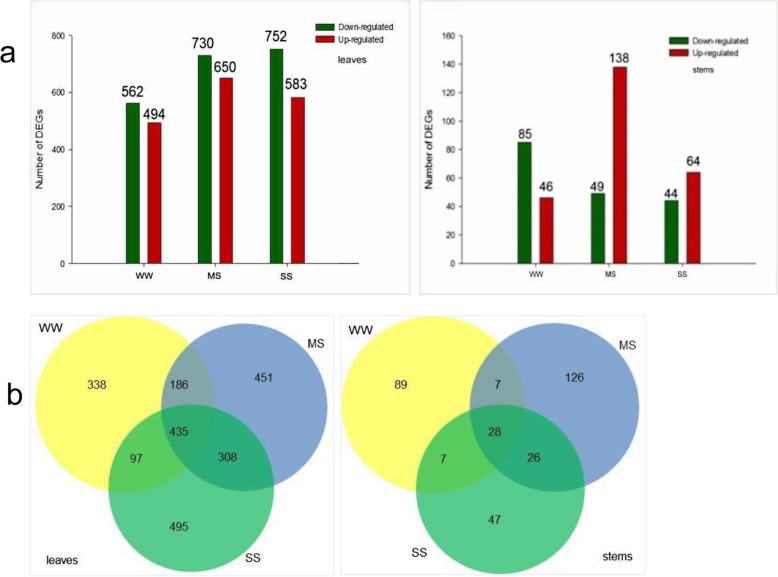
Analysis of differentially expressed genes (DEGs) between the non-transgenic maize line ZPM9 and the *SbER2–1*-overexpressing lines. **a** DEGs in leaves (left) and stems (right). **b** Wynn map analysis of DEGs in leaves (left) and stems (right)

To investigate the gene regulation network of the transgenic and NT lines under different drought treatments, we analyzed significant differentially expressed genes (DEGs) by RNA sequencing (RNA-Seq) (Fig. 6). In total, there were 1254 and 199 DEGs between the transgenic and NT lines under different conditions in the leaves and stems, respectively. In the leaves, we identified 1056 significant DEGs (false discovery rate (FDR) < 0.01, log_2_FC > 2) under WW conditions, and 1380 and 1335 significant DEGs under MS and SS conditions, respectively. In the stem, there were 126 DEGs under MS conditions, 47 DEGs under SS conditions, and 26 DEGs under MS and SS conditions. Thus, fewer DEGs were identified in stem than in leaf. Under WW, MS, and SS conditions, we identified 131, 187, and 108 significant DEGs, respectively. Venn diagrams of the DEGs under different drought stress conditions showed that there were 451 DEGs under MS conditions, 495 DEGs under SS conditions, and 308 common DEGs under both MS and SS conditions.

**Fig. 7:**
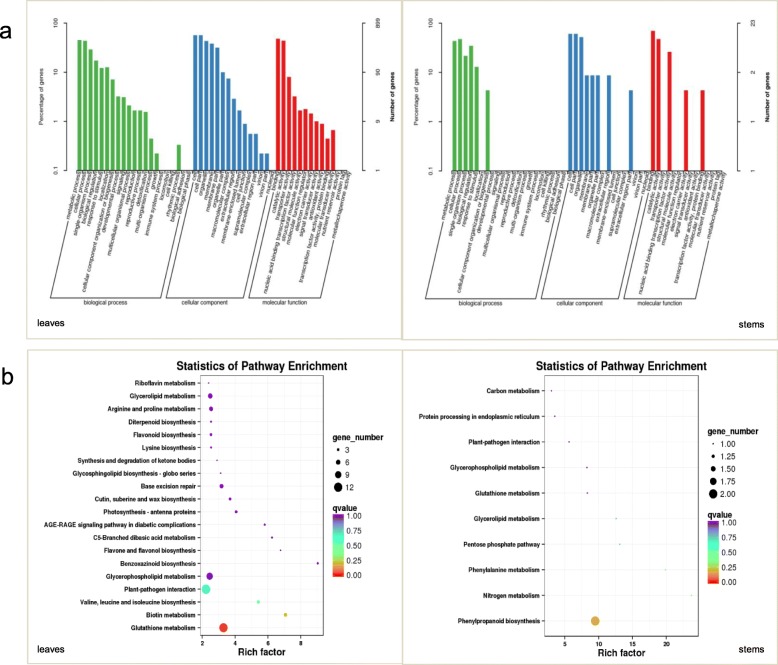
Functional enrichment analysis of DEGs between the non-transgenic ZPM9 and *SbER2–1* overexpression maize lines. **a** GO Functional enrichment analysis of DEGs in leaves (left) and stems (right). **b** KEGG pathway enrichment analysis of the pathways of DEGs in leaves (left) and stems (right)

Gene ontology (GO) analysis of the DEGs identified under drought conditions in the leaves and stems, revealed that the DEGs (both upregulated and downregulated) in both tissues were mainly enriched in metabolic processes, cellular processes, and response to stimulus in the biological process classification; cell parts, organelles, and membrane part in the cell composition classification; and binding function and catalytic activity in the molecular function classification. Next, we performed KEGG pathway enrichment analysis of DEGs between the transgenic and NT ZPM9 lines and between the leaves and stems (Fig. 7). In agreement with the large number of DEGs in the leaves, the number of enriched pathways was greater in the leaves than in the stems. The DEGs in the leaves were related mainly to 20 pathways, with the largest number related to the glutathione metabolic pathway, followed by the interaction of plant pathogens and glycerophospholipid metabolism. The DEGs in the stems were enriched in only ten related pathways, most of them involved in phenylpropanoid biosynthesis.

We focused on the candidate genes which enriched in glutathione metabolism in leaves and phenylpropanoid biosynthesis in stems. 12 DEGs were enriched in glutathione metabolism in leaves and only two DEGs were enriched in phenylpropanoid biosynthesis in stems. Among these, in leaves, *GRMZM2G028556* and *GRMZM2G032856*, which functions as glutathione transferases (GST) obtained by NCBI database, increased the expression levels in the *SbER2–1* transgenic maize lines compared with NT lines under severe drought stress. In stems, the expression levels of *GRMZM2G167613* and *GRMZM2G170692*, which functions as key enzyme of lignin synthetic pathway, cinnamyl alcohol dehydrogenase (CAD) and phenylalanine ammonia lyase (PAL) respectively, increased in the *SbER2–1* transgenic maize lines compared with NT lines under moderate drought stress. These results suggested that transgenic maize lines overexpressing *SbER2–1* may enhance drought tolerance involving in glutathione metabolism and phenylpropanoid biosynthesis.

### *SbER2–1* overexpression improves maize WUE under drought stress by increasing net photosynthetic rate

**Fig. 8:**
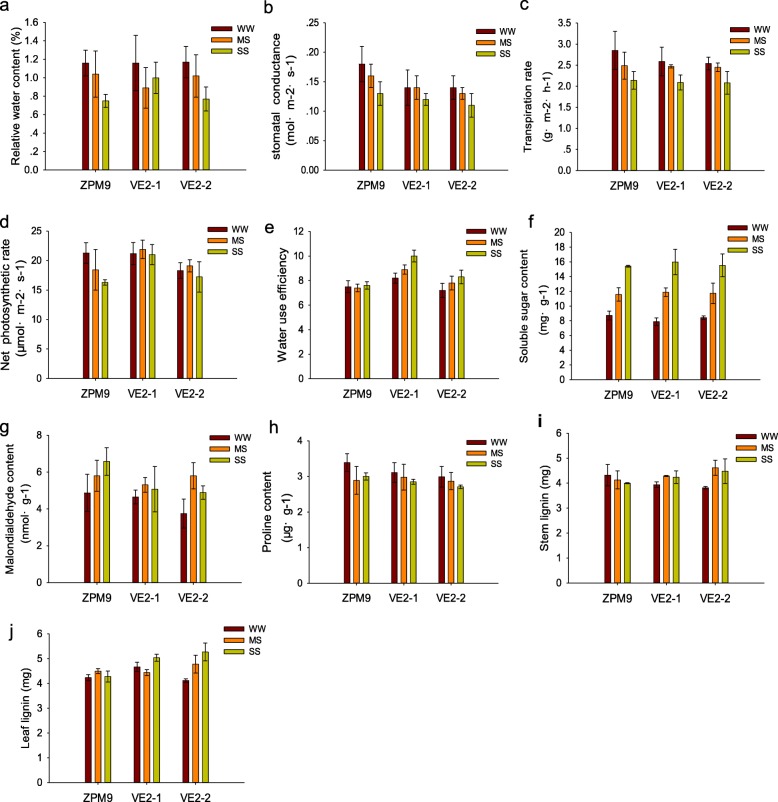
Phenotypic, physiological and biochemical characteristics of the *SbER2–1* overexpression maize lines VE2–1, VE2–2 and the non-transgenic receptor line ZPM9 under drought stress. **a** Leaf relative water content. **b**, **c**, **d**, **e** Physiological parameters related to photosynthesis and transpiration: stomatal conductance, transpiration rate, net photosynthetic rate and WUE. **f**, **g**, **h** Soluble sugar, MDA and proline contents. **i**, **j** Lignin contents in stems and leaves, respectively

To investigate the effect of *SbER2–1* on photosynthesis and transpiration traits, we analyzed the transgenic VE2–1 and VE2–2 and NT ZPM9 maize lines for the following characteristics: stomatal conductance, transpiration rate, net photosynthetic rate, and WUE (Fig. 8). There were no significant differences in stomatal conductance and transpiration rate between the transgenic and NT lines in response to drought stress. There also was no significant difference in net photosynthetic rate of the transgenic maize lines between the WW and drought treatments. However, the NT ZPM9 line showed significant decreases in net photosynthetic rate in response to drought stress, of 13.4 and 23.7% under MS and SS treatments, respectively (*P* < 0.05). The WUE of NT ZPM9 maize remained basically unchanged at the different water levels, while the WUE of the VE2–1 and VE2–2 transgenic plants increased significantly under MS and SS as compared with WW treatment (*P* < 0.01), with a greater increase observed in the *SbER2–1* transgenic line. These results indicated that the WUE of transgenic maize plants under drought stress was higher than that of the NT plants. These physiological phenotypes in the transgenic lines are consistent with a role for *SbER2–1* in promoting drought tolerance.

### *SbER2–1* transgenic plants increase lignin accumulation under drought stress

In this study, we also measured changes in proline, soluble sugar, and malondialdehyde (MDA) contents related to drought tolerance in plants. There was no significant difference in proline and soluble sugar contents between the transgenic and NT ZPM9 lines in response to drought stress (Fig. 8). There were also no significant differences in these parameters between the *SbER2–1*-overexpressing and NT ZPM9 plants in response to MS conditions, although the MDA content of *SbER2–1*-overexpressing plants under SS conditions was much lower than that of the NT ZPM9 line. Since MDA content reflects the degree of membrane peroxidation in plant cells, these findings indicated that *SbER2–1*-overexpressing plants have greater membrane antioxidant capacity. Upregulation of phenylpropanoid biosynthetic genes detected by RNA-Seq analysis of *SbER2–1*-overexpressing plants led us to measure lignin levels in the transgenic and NT maize lines (Fig. 8). Compared with NT ZPM9 plants, *SbER2–1*-overexpressing plants showed significantly higher lignin content in response to moderate and severe drought stress (*P* < 0.01).

## Discussion

### SbER is a protein kinase with LRR-RLK characteristics

RLKs are protein kinases that sense and transduce external stimuli and activate the expression of signaling factors. The protein encoded by the *Arabidopsis* gene *ER* is a LRR-RLK and is a signaling receptor subfamily member that regulates multiple signaling pathways [19]. In this study, we identified two *ER* homologs, *SbER1* and *SbER2*, from sorghum. Amino acid sequence analysis revealed very high sequence identity among the encoded proteins from several sorghum varieties, although the sequence identity for *SbER2* was lower than that for *SbER1*. *ER* possesses an unusual, characteristic exon–intron structure with 26 introns [11]. *SbER1* also has a typical LRR-RLK characteristic with 26 introns and is highly evolutionarily conserved, whereas the intron structure of *SbER2* did not conform this pattern. However, analysis of the sequence identity of *SbER2–1* from another sorghum variety, JZ12, revealed greater conservation of the LRR domain and conformation, with the typical 26 intron structures. It can be speculated that this difference may be the result of alternative *SbER2–1* mRNA splicing.

ER family receptors (ERs) are an ancient family of LRR-RLKs that in *Arabidopsis* consists of three genes: *ER*, *ERL1*, and *ERL2*. Phylogenetic analysis suggests that *ERL1* and *ERL2* have evolved through recent duplication, and are immediate paralogs of *ER* [13, 20]. In addition, molecular evolution studies have shown that the LRR-RLK subfamily in plants has undergone dramatic evolution. Studies have also shown that local genetic recombination or transformation events have occurred in the RLK subfamily during evolution, triggering intra-genetic functional differences and changing the position of homologous genes on chromosomes [21]. In this study, the coding amino acid sequence identity of *SbER1–1* and *SbER2–1* reached 77.9%. The bioinformatics analysis of the amino acid sequences encoded by *SbER1–1* and *SbER2–1* showed that both have the typical LRR-RLK protein kinase structure, which includes three domains: an extracellular leucine-rich receptor domain, a transmembrane domain, and an intracellular kinase domain [22]. Thus, we conclude that *SbER1* and *SbER2* may also be the result of gene duplication.

### *SbER2–1* confers drought tolerance in *Arabidopsis* and maize

In this study, *SbER* was expressed in stems and leaves, but not in roots, which is consistent with previous reports [23]. Under drought conditions, *SbER1* and *SbER2* were inducible and the expression levels of the two genes increased gradually with the severity of drought stress. *SbER2* exhibited stronger a response to drought compared with *SbER1*, especially in the stems, and was significantly upregulated under moderate and severe drought stress. Evaluation of the six sorghum varieties showed that drought tolerance increased with the level of *SbER2* expression, indicating that *SbER2* expression is closely associated with the drought stress response in sorghum. Additionally, *Arabidopsis* and maize lines overexpressing *SbER2–1* exhibited stronger drought tolerance than NT lines. Based on the high conservation of *ER* genes across species [24], we hypothesized that these genes positively regulate the drought stress response in sorghum. There are few studies on the relationship of *ER* to drought tolerance. Analysis of the 5′-terminal sequence of *PvER* genes from 145 wild and cultivated varieties of common beans (*Phaseolus vulgaris*) showed a range of adaptations to drought in wild beans that were not present in cultivated beans, which demonstrated an association between single-nucleotide polymorphisms (SNPs) in wild beans and drought tolerance [25]. A total of 11 *ER* gene SNPs are associated with different traits related to drought and heat tolerance in chickpea [26]. However, these studies only indicated an association between *ER* variation and drought tolerance based on a population analysis and lacked relevant expression evidence. In this study, the leaves of *SbER2–1*-overexpressing *Arabidopsis* plants remained green and fully expanded under drought stress, and *SbER2–1* transgenic maize seedlings had higher DTI compared with that of NT plants under severe drought stress. These findings indicate that *SbER2–1* overexpression is important in the maintenance of plant growth under drought conditions.

The net photosynthetic rate and WUE of *SbER2–1*-overexpressing maize plants increased with the extension of the severity and duration of drought conditions relative to that of NT plants. The net photosynthetic rate reflects the accumulation of organic matter in plants as a result of photosynthesis [27], and the plant WUE at the leaf level reflects the equilibrium between photosynthesis and transpiration. Prolonged exposure of plants to drought stress leads to destruction of the photosynthetic structure and inhibition of photosynthesis, with concomitant reduction in crop yield reduction [28, 29]. Anjum et al. (2011a) reported that drought stress in maize led to considerable decline in net photosynthesis, transpiration rate, stomatal conductance, WUE and intercellular CO_2_ compared to the well-watered control [30]. Masle et al. (2005) also reported that *ER* tends to maximize the electron transport and RuBisCO carboxylation capacity, which then affects the photosynthetic capacity in *Arabidopsis* [14]. A few studies have shown that in *Arabidopsis*, poplar *ER* family members are closely related to plant WUE, and overexpression of these genes improves the water-retention capacity of transgenic lines. The ER controls stomatal conductance and photosynthetic capacity in *Arabidopsis* and regulates plant development and control of WUE [14]. Overexpression of *PdER* from poplar in *Arabidopsis* significantly enhanced WUE by increasing the photosynthetic rate while reducing the transpiration rate [15]. Additionally, we showed in this study that the SbER2–1 protein is localized on the cell membrane and chloroplast. This suggests that it is involved in the synthesis of photosynthesis-related terpenoids, such as chlorophyll, carotenoids, and plastid quinones, thereby enhancing photosynthetic efficiency and WUE under drought stress conditions. Above all, we conclude that photosynthesis plays a very important role in *SbER2–1* transgenic plants and effectively improves plant maize WUE under drought stress. Thus, *SbER2–1* may have broad applicability in defending against drought stress in maize.

### Effects of transgenic *SbER2–1* on maize phenylpropanoid metabolism

In vivo, biological functions require the coordination of various genes, and pathway analysis further clarify the biological functions of genes. Through RNA-Seq and KEGG analysis of the responses of *SbER2–1*-overexpressing and NT plants to drought stress, we determined that the DEGs in stems were enriched in only ten related pathways, with phenylpropanoid biosynthesis being the most common. The phenylpropanoid metabolic pathway is an important secondary metabolic pathway in plants, as flavonoids, lignin, and other secondary substances produced by this pathway play crucial roles in plant stress resistance. Cruz et al. (1992) reported that under drought stress, the degree of lignification of sorghum roots is increased and the cell wall is thickened, which could limit the loss of internal tissue water and improve drought tolerance [31]. Similarly, grapevines respond to drought by modulating several secondary metabolic pathways by stimulating phenylpropanoid production in the grapes, potential influencing grape and wine antioxidant potential, composition, and sensory features [32].

Further analysis from phenylpropanoid biosynthesis revealed *GRMZM2G167613* and *GRMZM2G170692* were two key enzyme genes of lignin synthetic pathway, CAD and PAL that positively correlated with lignin content. Lignin can provide cell wall rigidity and enhance the ability of plant cells and tissues to resist other stresses [33, 34]. The increase of expression levels of *GRMZM2G167613* and *GRMZM2G170692* in transgenic maize lines may resist drought stress by raising the lignin content. Our analysis of lignin biosynthesis showed a higher lignin content in the transgenic *SbER2–1*-overexpressing plants than in NT ZPM9 under drought stress. Bang et al. (2019) also reported that overexpression of *OsTF1L*, a rice HD-Zip transcription factor, promotes lignin biosynthesis and stomatal closure that improve drought tolerance [35]. The results of this study suggest that the changes in the expression levels of multiple phenylpropanoid pathway genes in maize could, at least in part, explain the altered physiological characteristics of the transgenic plants. We therefore hypothesize that transgenic *SbER2–1* overexpression improves drought tolerance in maize by upregulating phenylpropanoid metabolism.

## Conclusions

Our results demonstrate that *SbER2–1* plays an important role in drought stress responses. Furthermore, photosynthetic systems and phenylpropanoid metabolism are implicated in *SbER2–1*-mediated drought stress tolerance mechanisms. Recent experience has shown that genetic engineering techniques to improve crops do not generally affect other beneficial traits [36–38]; thus, the *SbER2–1* gene represents an important candidate gene for use in genetic engineering technology to improve drought tolerance in maize.

## Methods

### Cultivation and treatment of sorghum materials

The seeds of JZ12 and six other sorghum varieties (10028D, V4A, Tx623B, Jinliangwu, 1383–2, and 363C/2691) were grown under greenhouse conditions at the Beijing Academy of Agricultural and Forestry Sciences (China). Seeds of uniform size and shape were sown in plastic pots (28 cm × 35 cm) with three holes for drainage in the bottom. To obtain good development, each pot was filled with total 8.0 kg of loamy soil, vermiculite, and nutrient soil mixed in a ratio of 1:1:1. Twenty seeds were sown in each pot, and three uniformly developed plants from each pot were selected at the 3-leaf stage.

We conducted an initial experiment to analyze *SbER* expression in response to drought stress. Plants at the 4-leaf stage were continuously subjected to water stress to set the soil moisture range. Plants were then watered to maintain a stable absolute soil water content that fluctuated within the ranges of 9–10%, 6–7%, and 17–18%, respectively, for WW conditions and two levels of drought conditions, moderate stress (MS) and severe stress (SS). Stress treatments were continued for 2 weeks, during which the absolute water content was determined using a soil moisture tester (TZS-1) and the last measurement was recorded. Following stress treatment, plants in one pot were removed from soil in their entirety and the roots were carefully cleaned of soil. The fresh weight (FW) was measured as the biomass, and then the plants were dried for 30 min in an oven at 105 °C, followed by 80 °C to a constant weight, which was recorded as the dry weight (DW). Finally, the roots, leaves, and stems of plants in the other pot were sampled, immediately frozen in liquid nitrogen and stored at − 80 °C for later analysis of *SbER* expression.

### Isolation of *SbER* and sequence analysis

Long fragment-specific amplification primers were designed according to the DNA and cDNA sequences of *SbER1* and *SbER2* in the NCBI database and the sorghum reference genome sequence (BTx623) (Additional file 3: Table S1). The sequences were then amplified using the LATaq enzyme (TaKaRa, Japan) using the following PCR reaction system (50 μL): 8 μL dNTPs (2.5 mM each); 0.5 μL LA Taq polymerase (5 U/μL); 5 μL 10× LA PCR Buffer (Mg^2+^ plus); 2 μL Primer-F (10 μM); 2 μL Primer-R (10 μM); 2 μL genomic DNA or cDNA template; and 30.5 μL distilled, deionized water (ddH_2_O). The DNA PCR procedure was as follows: 95 °C pre-denaturation, 5 min; 35× (95 °C denaturation, 40 s; 66 °C renaturation, 40 s; 72 °C extension, 8 min); 72 °C extension, 10 min. The same PCR protocol was used for generating cDNAs with the following modifications: 62 °C renaturation, 3 min; and 72 °C extension in the 35 cycles. A sample of the LAPCR product (5 μL) was separated by 1% agarose gel electrophoresis and, after the size was confirmed, the fragment of interest was cloned into the pEasyE1 vector (Transgen, China) for sequencing. The DNA, cDNA and deduced amino acid sequences were used to perform multiple alignment and phylogenetic analysis using the software DNAMAN 7.0. The evolutionary relationship between the members of the sorghum ER family and the *Arabidopsis*, maize, and rice ER family members was analyzed using the software MEGA 5.0.

### RNA isolation and qRT-PCR analysis

Total RNA was extracted from three tissue types (leaf (the top three), stem, and root) of four uniformly developed plants using an RNeasy Plant Mini Kit followed by the RNase-Free DNase Set (Qiagen, Hilden, Germany). The poly(A) mRNA was isolated from purified total RNA using oligo (dT) magnetic beads. The mRNA was fragmented into short pieces through addition of fragmentation buffer. The first-strand cDNA was synthesized by reverse transcriptase and random primers using mRNA fragments as templates. Second-strand cDNA synthesis was performed with RNase H and DNA polymerase I. Double-stranded cDNA fragments were subjected to end repair, 3′-dA tailing and adapter ligation. The required fragments were then purified and enriched by PCR to create the final cDNA library.

For qRT-PCR analysis, three biological replicates and three technical replicates were included. The qRT-PCR was performed using a Q6 Real-Time PCR System (Applied Biosystems, Foster City, CA, USA) according to the manufacturer’s instructions with the Maxima SYBR Green/ROX qPCR Master Mix (2×) (Thermo Scientific). All primers were designed using Primer Premier 5 (Premier Biosoft, Canada), and the sequences are listed in Additional file 4: Table S2. The primer specificity was validated by the identification of a single product-specific melting temperature in the melting profile. The PCR reaction system contained: 12.5 μl 2× real-time PCR mix, 1 μl gene-specific primers, 1 μl reverse transcribed cDNA product, and ddH_2_O. The thermal cycling program was as follows: 95 °C, 10 min, followed by 40 cycles of 95 °C, 15 s; 60 °C, 30 s; 72 °C, 30 s; and 82 °C, 5 s. At the end of the PCR cycles, melting-curve analysis was performed using a single cycle consisting of 95 °C, 15 s, and 60 °C, 1 min, followed by a slow temperature increase to 95 °C at the rate of 0.3 °C/s. The alpha-tubulin gene was used as the internal control to normalize the expression data. Relative expression levels were calculated according to the 2^-ΔΔCT^ (cycle threshold) method [39].

### Subcellular localization of SbER2–1

The ORF sequence of *SbER2–1* (without the stop codon) was amplified by PCR using the forward primer TTCTGCAGTCGACGGTACCATGGCCCGCCTCCTCCGGGC (containing the *Kpn*I restriction site) and reverse primer GCTTGTCTAGGATCCCGGGCTCCGTGCTTCGCGATATCAC (containing the *Xma*I restriction site). After digestion with *Kpn*I and *Xma*I, the fragment obtained was then cloned into the pE3449 vector, generating a construct (*35S::SbER2–1-eGFP*) that expresses *SbER2–1-eGFP* under the control of the CaMV *35S* promoter. The construct was confirmed by DNA sequencing. The pE3449 vector (expressing *35S::GFP*) and the *35S::SbER2–1-eGFP* construct were transformed into *Arabidopsis* mesophyll protoplast cells. Protoplast isolation and transfection were performed as described by Yoo et al. [40]. The transformed cells were incubated in the dark at 22 °C for 16 h and photographed using a laser-scanning confocal microscope (Zeiss LSM780).

### Plasmid construction and plant transformation

To overexpress the *SbER2–1* gene, we used the VK011 vector construction kit (Viewsolid Biotech). The full-length coding region of *SbER2–1* was amplified using a designed primer with a homologous linker (Additional file 3: Table S1). The target PCR product was extracted by gelation, and the concentration of the PCR product was quantified and diluted to 30 ng/μL for the recombinant ligation reaction. *Escherichia coli* DH5α were transformed with the recombinant product, and single recombinant clones were selected in LB liquid medium cultures containing kanamycin. The plasmid was extracted and double-digested with *Bam*HI and *Asc*I for preliminary detection and sequencing for further identification. The final construct, designated VK011-SbER2–1 (VE2), encoded the SbER2–1 protein expressed under the control of the maize ubiquitin promoter. This construct was introduced into *Agrobacterium tumefaciens* strains EHA105 and GV3101. *Arabidopsis* was transformed with *A. tumefaciens* strain GV3101 using the simplified floral-dip infiltration method [41], and the ZPM9 maize line was transformed with *A. tumefaciens* strain EHA105 according to the method described by Sidorov et al. (2009) [42]. The transformed plants were detected by PCR amplification using specifically designed primers (forward: TGATGGCATATGCAGCATCTATTC; reverse: AGATTCAGCCCAGAGAGGTTGA; amplification fragment length: 506 bp). The T_0_ generation positive plants were self-crossed, and T_1_ and T_2_ progeny were obtained by self-pollination after sowing. Homozygous transgenic lines were used for subsequent qRT-PCR and transcriptome analyses and drought tolerance assessment.

### Assessment of drought tolerance in transgenic lines

The transgenic and NT lines were subjected to drought to evaluate their drought tolerance capacity. Homozygous T_3_ seeds of transgenic *Arabidopsis* lines were used for phenotypic analysis of the effects of drought treatment. Positive transgenic lines and NT plants were sown in pots and watered regularly for 4 weeks. Then the seedlings were irrigated three times with equal volumes of ddH_2_O (control group) or 10% PEG 6000 solution (treatment group). About 2 weeks later the phenotypic states of the seedlings were evaluated.

For the transgenic and NT (ZPM9) maize lines, the experimental planting and drought treatment methods were the same as those used previously for evaluation of the sorghum varieties in this study. Using samples of the fully expanded leaf from the top of the plant, plant biomass, net photosynthetic rate, transpiration rate, leaf relative water content (RWC), and soluble sugar, proline, MDA, and lignin contents were measured after drought treatment. All measurements were repeated six times.

After 14 days of drought treatment, the FW of each plant was weighed and then the plant was placed in an oven for 30 min at 105 °C, followed by 80 °C to constant DW. For determination of the RWC of the leaves, the newest fully expanded leaf of each plant was excised and immediately weighed (FW). The leaves were then immersed in distilled water for 8 h, and then the surface moisture of the leaves was blotted with filter paper and the total weight (TW) was recorded. The DW was recorded after drying the leaves in an oven overnight at 80 °C. The leaf RWC was calculated as: (FW − DW)/(TW − DW) × 100%.

For measurement of leaf stomatal conductance, transpiration rate, and net photosynthetic rate, relevant parameters were determined using the topmost fully expanded leaf of the plant with a LI-6400XT portable photosynthesis meter. The photosynthetic photon flux density was set at 800 mmol m^− 2^ s^− 1^ with an internal 6400-02BLED source. All of the measurement were recorded between 9:30 and 11:00 in the morning.

Maize leaves (50 mg) were homogenized with deionized water, and soluble sugars were determined based on the phenol sulfuric acid method [43]. After boiling in water for 30 min, the total soluble sugars were extracted and absorbance at 620 nm determined using anthrone reagent with glucose as the standard. The soluble sugar content was then calculated.

Maize leaves (100 mg) were homogenized with deionized water, and free proline accumulation was determined according to the method described by Bates et al. (1973) [44]. Free proline was extracted with 100% methanol and 0.1% formic acid. Following centrifugation at 10 °C for 10 min, 1 mL of supernatant was transferred to a sample bottle and analyzed by liquid phase and mass spectrometry procedure.

MDA determination was done based on the method described by Quan et al. (2004) [45]. Maize leaves (100 mg) were homogenized with 3 ml extraction buffer and a small amount of quartz sand in an ice bath. The homogenate was subjected to ultrasound for 30 min and centrifuged, and then the MDA content of the supernatant was determined with 5% trichloroacetic acid (TCA) and 0.67% thiobarbituric acid (TBA).

Lignin content was determined from maize samples using the acetyl bromide method according to Terashima et al. (2009) [46].

### RNA-Seq analysis of transgenic maize

For maize RNA-Seq analysis, leaves and stems from three seedlings were collected from transgenic and NT ZPM9 lines exposed to WW, MS, and SS conditions. The total RNA was isolated from samples of ZPM9, VE2–1, and VE2–2 (three biological replicates). The Illumina sequencing was conducted by Biomarker Technologies Corporation using the HiSeq2500 system (Illumina Inc., USA). An average of 6.16 GB of raw data was generated for each sample. The RNA-Seq reads were mapped to maize reference genome B73 RefGen_v4 using TopHat [47, 48]. GO enrichment analysis was performed using agriGO v2.0 (http://bioinfo.cau.edu.cn/agriGO/).

## Supplementary information

**Additional file 1: Figure S1.** Amino acid sequence alignment of Arabidopsis, maize, rice and sorghum *ER* family gene. The online software BoxShade highlights different types of amino acid sequences: the black background represents a completely identical amino acid sequence and the gray background represents a similar amino acid sequence

**Additional file 2: Figure S2.** Analysis of *SbER2–1* sequencing results compared with *SbER2* in the NCBI database. The difference of 1-960 bp position of *SbER2–1* sequence was obvious compared with *SbER2*, and only 3 bp were substituted in 961-2683 bp position

**Additional file 3: Table S1.** The sequence of primers related to PCR amplication

**Additional file 4: Table S2.** The qRT-PCR primers of *SbER1–1*, *SbER2–1* and *alpha-tubulin*

## Data Availability

The data supporting the conclusions of this article are included within the article and its additional files.

## Abbreviations

DEG: differentially expressed gene
DTI: drought tolerance index
DW: dry weight
ER: ERECTA
FW: fresh weight
GO: Gene ontology
LRR-RLKs: leucine-rich repeat-type RLKs
MDA: malondialdehyde
MS: moderate stress
ORF: open reading frame
qRT-PCR: quantitative reverse transcribed polymerase chain reaction
RLCK: receptor-like cytoplasmic kinase
RLKs: Receptor-like protein kinases
RNA-Seq: RNA sequencing.
ROS: reactive oxygen species
RWC: relative water content
SDW: shoot dry weight
SFW: shoot fresh weight
S-RLKs: S-domain-type
SS: severe stress
TBA: thiobarbituric acid
TCA: trichloroacetic acid
TW: total weight
WUE: water use efficiency
WW: well-watered
