# Supplementary material for: A leucine-rich repeat-receptor-like kinase gene SbER2–1 from sorghum (Sorghum bicolor L.) confers drought tolerance in maize
Source: BMC Genomics. 2019 Oct 15;20:737. doi: 10.1186/s12864-019-6143-x (PMC6794760; doi:10.1186/s12864-019-6143-x)
Supplement: Supplementary file 1 — Additional file 1: Figure S1. Amino acid sequence alignment of Arabidopsis, maize, rice and sorghum ER family gene. The online software BoxShade highlights different types of amino acid sequences: the black background represents a completely identical amino acid sequence and the gray background represents a similar amino acid sequence [file 12864_2019_6143_MOESM1_ESM.docx]

*SbER1* 1 MPVRSSVAMTTTAARALVALLLV-----AVAVADD---------------------GATL
*SbER1-1* 1 MPVRSSVAMTTTAARALVALLLV-----AVAVADD---------------------GATL
*ZmER1* 1 MPVRSSVAMTTTAARALAALVLVTAAAAAAAVADD---------------------GAAL
*ZmER2* 1 MPVRSSVAMTTTAARALAALVLVTAAAAAAAVADD---------------------GAAL
*OsER1* 1 ----MTPAPAAASYRALVALLLV-----AVAVADD---------------------GSTL
*SbER2* 1 ---------MARLLRALAALLLLA----AAAVADDGWFPADFPCFGLVPAEVLLPGGATL
*SbER2-1* 1 ---------MARLLRALAALLLLA----AAAVADD---------------------GATL
*AtER* 1 ----------MALFRDIVLLGFLFCLSLVATVTSEE--------------------GATL
*AtER1* 1 --------MKEKMQRMVLSLAMVG--FMVFGVAS----AMNNE-------------GKAL
*OsER2* 1 ------MAAARAPWLWWWVVVVVG--VAVAEAASGGGGGGDGE-------------GKAL


*SbER1* 35 VEIKKSFRNVGNVLYDWAGD----DYCSWRGVLCDNVTFAVAALNLSGLNLEGEISPAVG
*SbER1-1* 35 VEIKKSFRNVGNVLYDWAGD----DYCSWRGVLCDNVTFAVAALNLSGLNLEGEISPAVG
*ZmER1* 40 VEIKKSFRNVGNVLYDWAGD----DYCSWRGVLCDNVTFAVAALNLSGLNLEGEISPAVG
*ZmER2* 40 VEIKKSFRNVGNVLYDWAGD----DYCSWRGVLCDNVTFAVAALNLSGLNLEGEISPAVG
*OsER1* 31 LEIKKSFRNVDNVLYDWAGG----DYCSWRGVLCDNVTFAVAALNLSGLNLGGEISPAVG
*SbER2* 48 LEIKKSFRDGGNALYDWSGDGASPGYCSWRGVLCDNVTFAVAAL----------------
*SbER2-1* 27 LEIKKSFRDGGNALYDWSGDGASPGYCSWRGVLCDNVTFAVAALNLSGLNLEGEISPAIG
*AtER* 31 LEIKKSFKDVNNVLYDWTTS-PSSDYCVWRGVSCENVTFNVVALNLSDLNLDGEISPAIG
*AtER1* 34 MAIKGSFSNLVNMLLDWDDV-HNSDLCSWRGVFCDNVSYSVVSLNLSSLNLGGEISPAIG
*OsER2* 40 MGVKAGFGNAANALVDWDG---GADHCAWRGVTCDNASFAVLALNLSNLNLGGEISPAIG


*SbER1* 91 SLKSLVSIDLKSNGLSGQIPDEIGDCSSLRTLDFSFNNLDGDIPFSISKLKHLENLILKN
*SbER1-1* 91 SLKSLVSIDLKSNGLSGQIPDEIGDCSSLRTLDFSFNNLDGDIPFSISKLKHLENLILKN
*ZmER1* 96 SLKSLVSIDLKSNGLSGQIPDEIGDCSSLRTLDFSFNNLDGDIPFSISKLKHLENLILKN
*ZmER2* 96 SLKSLVSIDLKSNGLSGQIPDEIGDCSSLRTLDFSFNNLDGDIPFSISKLKHLENLILKN
*OsER1* 87 RLKGIVSIDLKSNGLSGQIPDEIGDCSSLKTLDLSFNSLDGDIPFSVSKLKHIESLILKN
*SbER2* 92 --------DLKSNGLSGQIPDEIGDCSLLETLDLSSNNLEGDIPFSISKLKHLENLILKN
*SbER2-1* 87 SLQRVASIDLKSNGLSGQIPDEIGDCSLLETLDLSSNNLEGDIPFSISKLKHLENLILKN
*AtER* 90 DLKSLLSIDLRGNRLSGQIPDEIGDCSSLQNLDLSFNELSGDIPFSISKLKQLEQLILKN
*AtER1* 93 DLRNLQSIDLQGNKLAGQIPDEIGNCASLVYLDLSENLLYGDIPFSISKLKQLETLNLKN
*OsER2* 97 ELKNLQFVDLKGNKLTGQIPDEIGDCISLKYLDLSGNLLYGDIPFSISKLKQLEELILKN

*SbER1* 151 NQLIGAIPSTLSQLPNLKILDLAQNKLTGEIPRLIYWNEVLQYLGLRGNHLEGSLSPDMC
*SbER1-1* 151 NQLIGAIPSTLSQLPNLKILDLAQNKLTGEIPRLIYWNEVLQYLGLRGNHLEGSLSPDMC
*ZmER1* 156 NQLIGAIPSTLSQLPNLKILDLAQNKLTGEIPRLIYWNEVLQYLGLRGNHLEGSLSPDMC
*ZmER2* 156 NQLIGAIPSTLSQLPNLKILDLAQNKLTGEIPRLIYWNEVLQYLGLRGNHLEGSLSPDMC
*OsER1* 147 NQLIGVIPSTLSQLPNLKILDLAQNKLSGEIPRLIYWNEVLQYLGLRGNNLEGSISPDIC
*SbER2* 144 NNLVGVIPSTLSQLPNLKILDLAQNKLSGEIPNLIYWNEVLQYLGLRSNSLEGSLSSDMC
*SbER2-1* 147 NNLVGVIPSTLSQLPNLKILDLAQNKLSGEIPNLIYWNEVLQYLGLRSNSLEGSLSSDMC
*AtER* 150 NQLIGPIPSTLSQIPNLKILDLAQNKLSGEIPRLIYWNEVLQYLGLRGNNLVGNISPDLC
*AtER1* 153 NQLTGPVPATLTQIPNLKRLDLAGNHLTGEISRLLYWNEVLQYLGLRGNMLTGTLSSDMC
*OsER2* 157 NQLTGPIPSTLSQIPNLKTLDLAQNQLTGDIPRLIYWNEVLQYLGLRGNSLTGTLSPDMC

*SbER1* 211 QLTGLWYFDVKNNSLTGVIPDTIGNCTSFQVLDLSYNRFTGPIPFNIGFLQVATLSLQGN
*SbER1-1* 211 QLTGLWYFDVKNNSLTGVIPDTIRNCTSFQVLDLSYNRFTGPIPFNIGFLQVATLSLQGN
*ZmER1* 216 QLTGLWYFDVKNNSLTGAIPDTIGNCTSFQVLDLSYNRFTGPIPFNIGFLQVATLSLQGN
*ZmER2* 216 QLTGLWYFDVKNNSLTGAIPDTIGNCTSFQVLDLSYNRFTGPIPFNIGFLQVATLSLQGN
*OsER1* 207 QLTGLWYFDVKNNSLTGPIPETIGNCTSFQVLDLSYNKLSGSIPFNIGFLQVATLSLQGN
*SbER2* 204 QLTGLWY-----------------------------------------------LSLQGN
*SbER2-1* 207 QLTGLWYFDVKNNSLTGTVPETIGNCTSFQVLDLSNNHLTGEIPFNIGFLQVATLSLQGN
*AtER* 210 QLTGLWYFDVRNNSLTGSIPETIGNCTAFQVLDLSYNQLTGEIPFDIGFLQVATLSLQGN
*AtER1* 213 QLTGLWYFDVRGNNLTGTIPESIGNCTSFQILDISYNQITGEIPYNIGFLQVATLSLQGN
*OsER2* 217 QLTGLWYFDVRGNNLTGTIPESIGNCTSFEILDISYNQISGEIPYNIGFLQVATLSLQGN

*SbER1* 271 KFTGPIPSVIGLMQALAVLDLSYNQLSGPIPSILGNLTYTEKLYIQGNKLTGSIPPELGN
*SbER1-1* 271 KFTGPIPSVIGLMQALAVLDLSYNQLSGPIPSILGNLTYTEKLYIQGNKLTGSIPPELGN
*ZmER1* 276 KFTGPIPSVIGLMQALAVLDLSYNQLSGPIPSILGNLTYTEKLYMQGNRLTGSIPPELGN
*ZmER2* 276 KFTGPIPSVIGLMQALAVLDLSYNQLSGPIPSILGNLTYTEKLYMQGNRLTGSIPPELGN
*OsER1* 267 MFTGPIPSVIGLMQALAVLDLSYNQLSGPIPSILGNLTYTEKLYMQGNKLTGPIPPELGN
*SbER2* 217 KFSGPIPSVIGLMQALAVLDLSFNELSGPIPSILGNLTYTEK------------------
*SbER2-1* 267 KFSGPIPSVIGLMQALAVLDLSFNELSGPIPSILGNLTYTEKLYLQGNRLTGSIPPELGN
*AtER* 270 QLSGKIPSVIGLMQALAVLDLSGNLLSGSIPPILGNLTFTEKLYLHSNKLTGSIPPELGN
*AtER1* 273 RLTGRIPEVIGLMQALAVLDLSDNELVGPIPPILGNLSFTGKLYLHGNMLTGPIPSELGN
*OsER2* 277 RLTGKIPDVIGLMQALAVLDLSENELVGPIPSILGNLSYTGKLYLHGNKLTGVIPPELGN

*SbER1* 331 MSTLHYLELNDNQLTGSIPPELGRLTGLFDLNLANNHLEGPIPDNLSSCVNLNSFNAYGN
*SbER1-1* 331 MSTLHYLELNDNQLTGSIPPELGRLTGLFDLNLANNHLEGPIPDNLSSCVNLNSFNAYGN
*ZmER1* 336 MSTLHYLELNDNQLTGSIPPELGRLTGLFDLNLANNHLEGPIPDNLSSCVNLNSFNAYGN
*ZmER2* 336 MSTLHYLELNDNQLTGSIPPELGRLTGLFDLNLANNHLEGPIPDNLSSCVNLNSFNAYGN
*OsER1* 327 MSTLHYLELNDNQLSGFIPPEFGKLTGLFDLNLANNNFEGPIPDNISSCVNLNSFNAYGN
*SbER2* 259 ------LELNDNLLTGFIPPDLGKLTELFELNLANNNLIGPIPENLSSCANLIS------
*SbER2-1* 327 MSTLHYLELNDNLLTGFIPPDLGKLTELFELNLANNNLIGPIPENLSSCANLISFNAYGN
*AtER* 330 MSKLHYLELNDNHLTGHIPPELGKLTDLFDLNVANNDLEGPIPDHLSSCTNLNSLNVHGN
*AtER1* 333 MSRLSYLQLNDNKLVGTIPPELGKLEQLFELNLANNRLVGPIPSNISSCAALNQFNVHGN
*OsER2* 337 MSKLSYLQLNDNELVGTIPAELGKLEELFELNLANNNLQGPIPANISSCTALNKFNVYGN

*SbER1* 391 KLNGTIPRSLRKLESMTYLNLSSNFISGSIPIELSRINNLDTLDLSCNMMTGPIPSSIGS
*SbER1-1* 391 KLNGTIPRSLRKLESMTYLNLSSNFISGSIPIESSRINNLDTLDLSCNMMTGPIPSSIGS
*ZmER1* 396 KLNGTIPRSLRKLESMTYLNLSSNFISGSIPIELSRINNLDTLDLSCNMMTGPIPSSIGN
*ZmER2* 396 KLNGTIPRSLRKLESMTYLNLSSNFISGSIPIELSRINNLDTLDLSCNMMTGPIPSSIGN
*OsER1* 387 RLNGTIPPSLHKLESMTYLNLSSNFLSGSIPIELSRINNLDTLDLSCNMITGPIPSTIGS
*SbER2* 307 ------------------LNLSSNHLSGALPIEVARMRNLDTLDLSCNMITGSIPSAIGK
*SbER2-1* 387 KLNGTIPRSFHKLESLTYLNLSSNHLIGALPIEVARMRNLDTLDLSCNMITGSIPSAIGK
*AtER* 390 KFSGTIPRAFQKLESMTYLNLSSNNIKGPIPVELSRIGNLDTLDLSNNKINGIIPSSLGD
*AtER1* 393 LLSGSIPLAFRNLGSLTYLNLSSNNFKGKIPVELGHIINLDKLDLSGNNFSGSIPLTLGD
*OsER2* 397 KLNGSIPAGFQKLESLTYLNLSSNNFKGNIPSELGHIINLDTLDLSYNEFSGPVPATIGD

*SbER1* 451 LEHLLRLNLSKNGLVGFIPAEFGNLRSVMEIDLSYNHLGGLIPQELEMLQNLMLLKLENN
*SbER1-1* 451 LEPLLRLNLSKNGLVGFIPAGFGNLRSVMEIDLSYNHLGGLIPQELEMLQNLMLLKLENN
*ZmER1* 456 LEHLLRLNLSKNDLVGFIPAEFGNLRSVMEIDLSYNHLGGLIPQELGMLQNLMLLKLENN
*ZmER2* 456 LEHLLRLNLSKNDLVGFIPAEFGNLRSVMEIDLSYNHLGGLIPQELGMLQNLMLLKLENN
*OsER1* 447 LEHLLRLNLSNNGLVGFIPAEIGNLRSIMEIDMSNNHLGGLIPQELGMLQNLMLLNLKNN
*SbER2* 349 LEHLLRLNLSKNNVGGHIPAEFGNLRSIMEIDLSYNHLLGLIPQEVGMLQNLILLKLESN
*SbER2-1* 447 LEHLLRLNLSKNNVGGHIPAEFGNLRSIMEIDLSYNHLLGLIPQEVGMLQNLILLKLESN
*AtER* 450 LEHLLKMNLSRNHITGVVPGDFGNLRSIMEIDLSNNDISGPIPEELNQLQNIILLRLENN
*AtER1* 453 LEHLLILNLSRNHLSGQLPAEFGNLRSIQMIDVSFNLLSGVIPTELGQLQNLNSLILNNN
*OsER2* 457 LEHLLELNLSKNHLDGPVPAEFGNLRSVQVIDMSNNNLSGSLPEELGQLQNLDSLILNNN

*SbER1* 511 NITGDLSS-LMNCFSLNILN---VSYNNLAGVVPADNNFTRFSP----------------
*SbER1-1* 511 NITGDLSS-LMNCFSLNILN---VSYNNLAGVVPADNNFTRFSP----------------
*ZmER1* 516 NITGDVSS-LMNCFSLNILN---VSYNNLAGAVPTDNNFTRFSH----------------
*ZmER2* 516 NITGDVSS-LMNCFSLNILN---VSYNNLAGAVPTDNNFTRFSH----------------
*OsER1* 507 NITGDVSS-LMNCFSLNILN---VSYNNLAGVVPTDNNFSRFSP----------------
*SbER2* 409 NITGDVSS-LAYCLSLNVLN---VSYNHLYGIVPTDNNFSRFSP----------------
*SbER2-1* 507 NITGDVSS-LAYCLSLNVLN---VSYNHLYGIVPTDNNFSRFSP----------------
*AtER* 510 NLTGNVGS-LANCLSLTVLN---VSHNNLVGDIPKNNNFSRFSP----------------
*AtER1* 513 KLHGKIPDQLTNCFTLVNLN---VSFNNLSGIVPPMKNFSRFAP----------------
*OsER2* 517 NLVGEIPAQLANCFSLNNLAFQEFVIQQFIWTCPDGKELLEIPNGKHLLISDCNQYINHK

*SbER1* 551 DSFLGNPGLCGYWLG-SSCRSTGHHEKPPIS------KAAIIGVAVGGLVILLMILVAVC
*SbER1-1* 551 DSFLGNPGLCGYWLG-SSCRSTGHHEKPPIS------KAAIIGVAVGGLVILLMILVAVC
*ZmER1* 556 DSFLGNPGLCGYWLG-SSCRSTGHRDKPPIS------KAAIIGVAVGGLVILLMILVAVC
*ZmER2* 556 DSFLGNPGLCGYWLG-SSCRSTGHRDKPPIS------KAAIIGVAVGGLVILLMILVAVC
*OsER1* 547 DSFLGNPGLCGYWLG-SSCRSSGHQQKPLIS------KAAILGIAVGGLVILLMILVAVC
*SbER2* 449 DSFLGNPGLCGYWLRSSSCTQLPSAEKMKTSSTSKAPKAAFIGIGVVGLVILLVILVAVC
*SbER2-1* 547 DSFLGNPGLCGYWLRSSSCTQLPSAEKMKTSSTSKAPKAAFIGIGVVGLVILLVILVAVC
*AtER* 550 DSFIGNPGLCGSWLN-SPCHDSRRTVRVSIS------RAAILGIAIGGLVILLMVLIAAC
*AtER1* 554 ASFVGNPYLCGNWVG-SICGPLPK-SRV-FS------RGALICIVLGVITLLCMIFLAVY
*OsER2* 577 CSFLGNPLLHVYCQD-SSCGHSHG-QRVNIS------KTAIACIILGFIILLCVLLLAIY


*SbER1* 604 RPHRPPAFKDVTVSKP------VRNAPPKLVILHMNMALHVYDDIMRMTENLSEKYIIGY
*SbER1-1* 604 RPHRPPAFKDVTVSKP------VRNAPPKLVILHMNMALHVYDDIMRMTENLSEKYIIGY
*ZmER1* 609 RPHHPPAFKDATVSKP------VSNGPPKLVILHMNMALHVFDDIMRMTENLSEKYIIGY
*ZmER2* 609 RPHHPPAFKDATVSKP------VSNGPPKLVILHMNMALHVFDDIMRMTENLSEKYIIGY
*OsER1* 600 RPHSPPVFKDVSVSKP------VSNVPPKLVILHMNLSLLVYEDIMTMTENLSEKYIIGY
*SbER2* 509 WPQNSPVPKDVSVNKPDNLAAASSNVPPKLVILHMNMALHVYDDIMRMTENLSEKYIIGY
*SbER2-1* 607 WPQNSPVPKDVSVNKPDNLAAASSNVPPKLVILHMNMALHVYDDIMRMTENLSEKYIIGY
*AtER* 603 RPHNPPPFLDGSLDKP------VTYSTPKLVILHMNMALHVYEDIMRMTENLSEKYIIGH
*AtER1* 605 KSMQQKKILQGS-SKQ-------AEGLTKLVILHMDMAIHTFDDIMRVTENLNEKFIIGY
*OsER2* 629 KTNQPQPLVKGS-DKP-------VQGPPKLVVLQMDMAIHTYEDIMRLTENLSEKYIIGY

**Signal seq**

**LRR domain**

**Kinase domain**

**transmembrane domain**

*SbER1* 658 GASSTVYKCVLKNCKPVAIKKLYAHYPQSLKEFETELETVGSIKHRNLVSLQGYSLSPVG
*SbER1-1* 658 GASSTVYKCVLKNCKPVAIKKLYAHYPQSLKEFETELETVGSIKHRNLVSLQGYSLSPVG
*ZmER1* 663 GASSTVYKCVLKNCKPVAIKKLYAHYPQSLKEFETELETVGSIKHRNLVSLQGYSLSPVG
*ZmER2* 663 GASSTVYKCVLKNCKPVAIKKLYAHYPQSLKEFETELETVGSIKHRNLVSLQGYSLSPVG
*OsER1* 654 GASSTVYKCVSKNRKPVAVKKLYAHYPQSFKEFETELETVGSIKHRNLVSLQGYSLSPVG
*SbER2* 569 GASSTVYRCDLKNCKPIAIKKLYAHYPQSLKEFETELETVGSIKHRNLVSLQGYSLSPSG
*SbER2-1* 667 GASSTVYRCDLKNCKPIAIKKLYAHYPQSLKEFETELETVGSIKHRNLVSLQGYSLSPSG
*AtER* 657 GASSTVYKCVLKNCKPVAIKRLYSHNPQSMKQFETELEMLSSIKHRNLVSLQAYSLSHLG
*AtER1* 657 GASSTVYKCALKSSRPIAIKRLYNQYPHNLREFETELETIGSIRHRNIVSLHGYALSPTG
*OsER2* 681 GASSTVYKCELKSGKAIAVKRLYSQYNHSLREFETELETIGSIRHRNLVSLHGFSLSPHG

*SbER1* 718 NLLFYDYMECGSLWDVLHEGSSKKKKLDWETRLRIALGAAQGLAYLHHDCSPRIIHRDVK
*SbER1-1* 718 NLLLYDYMECGSLWDVLHEGSSKKKKLDWETRLRIALGAAQGLAYLHHDCSPRIIHRDVK
*ZmER1* 723 NLLFYDYMESGSLWDVLHEGSSKKNKLDWVTRLRIALGAAQGLAYLHHDCSPRIIHRDVK
*ZmER2* 723 NLLFYDYMESGSLWDVLH-GSSKKNKLDWVTRLRIALGAAQGLAYLHHDCSPRIIHRDVK
*OsER1* 714 NLLFYDYMENGSLWDVLHEGPTKKKKLDWETRLRIALGAAQGLAYLHHDCSPRIIHRDVK
*SbER2* 629 NLLFYDYLENGSLWDILHAASSKKKKLDWEARLKIALGAAHGLAYLHHECSPRIIHRDVK
*SbER2-1* 727 NLLFYDYLENGSLWDILHAASSKKKKLDWEARLKIALGAAHGLAYLHHGCSPRIIHRDVK
*AtER* 717 SLLFYDYLENGSLWDLLH-GPTKKKTLDWDTRLKIAYGAAQGLAYLHHDCSPRIIHRDVK
*AtER1* 717 NLLFYDYMENGSLWDLLH-GSLKKVKLDWETRLKIAVGAAQGLAYLHHDCTPRIIHRDIK
*OsER2* 741 NLLFYDYMENGSLWDLLH-GPSKKVKLNWDTRLRIAVGAAQGLAYLHHDCNPRIIHRDVK

*SbER1* 778 SKNILLDKDYEAHLTDFGIAKSLCVSKTHTSTYVMGTIGYIDPEYARTSRLNEKSDVYSY
*SbER1-1* 778 SKNVLLDKDYEAHLTDFGIAKSLCVSKTHTSTYVMGTIGYIDPEYARTSRLNEKSDVYSY
*ZmER1* 783 SKNILLDKDYEAHLTDFGIAKSLCVSKTHTSTYVMGTIGYIDPEYARTSRLNEKSDVYSY
*ZmER2* 782 SKNILLDKDYEAHLTDFGIAKSLCVSKTHTSTYVMGTIGYIDPEYARTSRLNEKSDVYSY
*OsER1* 774 SKNILLDKDYEAHLTDFGIAKSLCVSKTHTSTYVMGTIGYIDPEYARTSRLNEKSDVYSY
*SbER2* 689 SKNILLDKDYEAHLADFGIAKSLCVSKTHTSTYVMGTIGYIDPEYARTSRLNEKSDVYSY
*SbER2-1* 787 SKNILLDKDYEAHLADFGIAKSLCVSKTHTSTYVMGTIGYIDPEYARTSRLNEKSDVYSY
*AtER* 776 SSNILLDKDLEARLTDFGIAKSLCVSKSHTSTYVMGTIGYIDPEYARTSRLTEKSDVYSY
*AtER1* 776 SSNILLDENFEAHLSDFGIAKSIPASKTHASTYVLGTIGYIDPEYARTSRINEKSDIYSF
*OsER2* 800 SSNILLDENFEAHLSDFGIAKCVPSAKSHASTYVLGTIGYIDPEYARTSRLNEKSDVYSF

*SbER1* 838 GIVLLELLTGKKPVDNECNLHHLILSKTASNEVMDTVDPDIGDTCKDLGEVKKLFQLALL
*SbER1-1* 838 GIVLLELLTGKKPVDNECNLHHLILSKTAGNEVMDTVDPDIGDTCKDLGEVKKLFQLALL
*ZmER1* 843 GIVLLELLTGKKPVDNECNLHHLILSKTASNEVMETVDPDVGDTCKDLGEVKKLFQLALL
*ZmER2* 842 GIVLLELLTGKKPVDNECNLHHLILSKTASNEVMETVDPDVGDTCKDLGEVKKLFQLALL
*OsER1* 834 GIVLLELLTGKKPVDNECNLHHLILSKTANNAVMETVDPDIADTCKDLGEVKKVFQLALL
*SbER2* 749 GIVLLELLTGKKPVDDECNLHHLILSKAAENTVMEMVDQDITDTCKDLGEVKKVFQLALL
*SbER2-1* 847 GIVLLELLTGKKPVDDECNLHHLILSKAAENTVMEMVDQDITDTCKDLGEVKKMFQLALL
*AtER* 836 GIVLLELLTRRKAVDDESNLHHLIMSKTGNNEVMEMADPDITSTCKDLGVVKKVFQLALL
*AtER1* 836 GIVLLELLTGKKAVDNEANLHQLILSKADDNTVMEAVDPEVTVTCMDLGHIRKTFQLALL
*OsER2* 860 GIVLLELLTGKKAVDNESNLHQLILSKADDNTVMEAVDSEVSVTCTDMGLVRKAFQLALL

*SbER1* 898 CTKRQPSDRPTMHEVVRVLDCLVNPDPPPKPS-------AHQLPQPSPAVPSYINEYVSL
*SbER1-1* 898 CTKRQPSDRPTMHEVVRVLDCLVNPDPPPKPS-------AHQLPQPSPAVPSYINEYVSL
*ZmER1* 903 CTKRQPSDRPTMHEVVRVLDCLVNPEPPPQPQQQQQKAHAHHQLPPQPSPPAYVDEYVSL
*ZmER2* 902 CTKRQPSDRPTMHEVVRVLDCLVNPEPPPQPQQQQQKAHAHHQLPPQPSPPAYVDEYVSL
*OsER1* 894 CTKRQPSDRPTMHEVVRVLDCLVRPDPPPKSA-------QQLAMPQRPAVPSYINEYVSL
*SbER2* 809 CSKRQPSDRPTMHEVARVLDSLVCPGPLPKQA-------QPQALEKSSTAPSYVSEYVGL
*SbER2-1* 907 CSKRQPSDRPTMHEVARVLDSLVCPGPLPKQA-------QPQALEKSSTAPSYVSEYVGL
*AtER* 896 CTKRQPNDRPTMHQVTRVLGSFMLSEQPPAAT----------DTSATLAGSCYVDEYANL
*AtER1* 896 CTKRNPLERPTMLEVSRVLLSLVPSLQVAK------------------KLPSLDHSTKKL
*OsER2* 920 CTKRHPSDRPTMHEVARVLLSLLPASAMTTPK-----------TVDYSRLLASTTTAADM

**C-terminal tail region**

*SbER1* 951 RGTGALSCANSTSTSDAELFLKFGEAISQNME
*SbER1-1*  951 RGTGALSCANSTSTSDAELFLKFGEAISQNME
*ZmER1* 963 RGTGALSCANSSSTSDAELFLKFGEAISQNMV
*ZmER2* 962 RGTGALSCANSSSTSDAELFLKFGEAISQNMV
*OsER1* 947 RGTSVLSCANSSCTSDAELFLKFGEVISQNTE
*SbER2* 862 RGGSALSCANSSSASDAELFMKFGEVISRSTE
*SbER2-1* 960 RGGSALSCANSSSASDAELFMKFGEVISRSTE
*AtER* 946 KTPHSVNCS-SMSASDAQLFLRFGQVISQNSE
*AtER1* 938 Q---QENEVRNPDAEASQWFVQFREVISKSSI
*OsER2* 969 RG-HDVTDIGDNSSSDEQWFVRFGEVISKHTM

**Additional file 1: Fig. S1** Amino acid sequence alignment of Arabidopsis, maize, rice and sorghum *ER* family gene. The online software BoxShade highlights different types of amino acid sequences: the black background represents a completely identical amino acid sequence and the gray background represents a similar amino acid sequence.
