# Supplementary material for: A leucine-rich repeat-receptor-like kinase gene SbER2–1 from sorghum (Sorghum bicolor L.) confers drought tolerance in maize
Source: BMC Genomics. 2019 Oct 15;20:737. doi: 10.1186/s12864-019-6143-x (PMC6794760; doi:10.1186/s12864-019-6143-x)
Supplement: Supplementary file 2 — Additional file 2: Figure S2. Analysis of SbER2–1 sequencing results compared with SbER2 in the NCBI database. The difference of 1-960 bp position of SbER2–1 sequence was obvious compared with SbER2, and only 3 bp were substituted in 961-2683 bp position [file 12864_2019_6143_MOESM2_ESM.docx]

SbER2-1 1 ATGGCCCGCCTCCTCCGGGCCCTCGCCGCCCTCCTCCTCCTCGCGGCCGCCGCCGTCGCC
*SbER2* 1 ATGGCCCGCCTCCTCCGGGCCCTCGCCGCCCTCCTCCTCCTCGCGGCCGCCGCCGTCGCC

SbER2-1 61 GACGACGG----------------------------------------------------
*SbER2* 61 GACGACGGTTGGTTCCCCGCCGACTTCCCGTGCTTCGGTTTGGTTCCCGCAGAGGTTTTG

SbER2-1 69 -----------GGCAACACTGCTGGAGATCAAGAAATCCTTCCGCGACGGCGGCAACGCG
*SbER2* 121 CTCCCTGGTGGGGCAACACTGCTGGAGATCAAGAAATCCTTCCGCGACGGCGGCAACGCG

SbER2-1 118 CTGTACGATTGGTCCGGCGATGGCGCATCGCCGGGCTACTGCTCGTGGCGCGGCGTGCTA
*SbER2* 181 CTGTACGATTGGTCCGGCGATGGCGCATCGCCGGGCTACTGCTCGTGGCGCGGCGTGCTA

SbER2-1 178 TGCGACAACGTCACCTTCGCTGTCGCCGCGCTCAACCTCTCTGGGCTGAATCTCGAGGGT
*SbER2* 241 TGCGACAACGTCACCTTCGCTGTCGCCGCGCT----------------------------

SbER2-1 238 GAAATCTCACCGGCCATCGGGAGTCTGCAACGTGTTGCCTCAATAGATTTGAAGTCGAAT
*SbER2* 273 --------------------------------------------AGATTTGAAGTCGAAT

SbER2-1 298 GGACTCTCGGGACAGATCCCTGATGAGATTGGTGATTGTTCGTTGCTTGAAACTCTGGAC
*SbER2* 289 GGACTCTCGGGACAGATCCCTGATGAGATTGGTGATTGTTCGTTGCTTGAAACTCTGGAC

SbER2-1 358 TTGTCCTCTAACAATCTAGAAGGAGACATACCATTCTCCATATCCAAGCTGAAGCACCTT
*SbER2* 349 TTGTCCTCTAACAATCTAGAAGGAGACATACCATTCTCCATATCCAAGCTGAAGCACCTT

SbER2-1 418 GAGAACTTGATTTTGAAGAACAACAATCTGGTGGGGGTGATTCCATCGACACTCTCTCAA
*SbER2* 409 GAGAACTTGATTTTGAAGAACAACAATCTGGTGGGGGTGATTCCATCGACACTCTCTCAA

SbER2-1 478 CTTCCAAATTTGAAGATATTGGACTTGGCTCAAAACAAGCTAAGTGGTGAAATTCCAAAT
*SbER2* 469 CTTCCAAATTTGAAGATATTGGACTTGGCTCAAAACAAGCTAAGTGGTGAAATTCCAAAT

SbER2-1 538 CTAATATATTGGAATGAGGTTCTTCAATACTTGGGATTGCGAAGCAATAGTTTAGAAGGA
*SbER2* 529 CTAATATATTGGAATGAGGTTCTTCAATACTTGGGATTGCGAAGCAATAGTTTAGAAGGA

SbER2-1 598 AGCCTATCTTCCGATATGTGCCAGTTAACTGGTCTGTGGTACTTTGATGTGAAGAACAAT
*SbER2* 589 AGCCTATCTTCCGATATGTGCCAGTTAACTGGTCTGTGGTACTT----------------

SbER2-1 658 AGCTTGACGGGTACAGTACCAGAAACCATAGGGAACTGTACAAGCTTTCAGGTCTTGGAT
*SbER2* 633 ------------------------------------------------------------

SbER2-1 718 TTGTCAAACAATCACCTTACTGGAGAAATCCCATTCAATATTGGTTTCCTGCAAGTGGCT
*SbER2* 633 ------------------------------------------------------------

SbER2-1 778 ACTTTGTCTTTGCAAGGGAACAAATTCTCTGGCCCTATACCATCAGTGATTGGCCTTATG
*SbER2* 633 -----GTCTTTGCAAGGGAACAAATTCTCTGGCCCTATACCATCAGTGATTGGCCTTATG

SbER2-1 838 CAGGCGCTTGCAGTGCTGGATCTGAGTTTCAATGAGCTATCGGGCCCAATACCGTCTATA
*SbER2* 688 CAGGCGCTTGCAGTGCTGGATCTGAGTTTCAATGAGCTATCGGGCCCAATACCGTCTATA

SbER2-1 898 CTGGGCAACTTGACATACACTGAGAAATTATACCTGCAAGGCAACAGGCTAACTGGATCG
*SbER2* 748 CTGGGCAACTTGACATACACTGAGAAATT-------------------------------

SbER2-1 958 ATACCACCAGAGCTTGGTAATATGTCGACACTGCATTACCTGGAACTGAACGACAATCTA
*SbER2* 777 -----------------------------------------GGAACTGAACGACAATCTA

SbER2-1 1018 TTGACTGGGTTCATTCCTCCTGATCTTGGAAAACTCACAGAATTGTTTGAATTGAACCTT
*SbER2* 796 TTGACTGGGTTCATTCCTCCTGATCTTGGAAAACTCACAGAATTGTTTGAATTGAACCTT

SbER2-1 1078 GCAAACAACAACCTTATAGGGCCTATCCCTGAGAATTTAAGTTCATGTGCAAATCTCATT
*SbER2* 856 GCAAACAACAACCTTATAGGGCCTATCCCTGAGAATTTAAGTTCATGTGCAAATCTCATT

SbER2-1 1138 AGTTTCAATGCTTATGGCAATAAATTGAATGGAACCATTCCACGTTCATTTCACAAGCTT
*SbER2* 916 AGTTT-------------------------------------------------------

SbER2-1 1198 GAGAGTCTGACTTATCTGAATCTGTCATCAAATCATCTCATTGGAGCACTTCCAATTGAG
*SbER2* 921 -----------------GAATCTGTCATCAAATCATCTCAGTGGAGCACTTCCAATTGAG

**Additional file 2: Fig. S2** Analysis of *SbER2-1* sequencing results compared with *SbER2* in the NCBI database. The difference of 1-960bp position of *SbER2-1* sequence was obvious compared with *SbER2*, and only 3 bp were substituted in 961-2683bp position.
