# Supplementary material for: A leucine-rich repeat-receptor-like kinase gene SbER2–1 from sorghum (Sorghum bicolor L.) confers drought tolerance in maize
Source: BMC Genomics. 2019 Oct 15;20:737. doi: 10.1186/s12864-019-6143-x (PMC6794760; doi:10.1186/s12864-019-6143-x)
Supplement: Supplementary file 3 — Additional file 3: Table S1. The sequence of primers related to PCR amplication [file 12864_2019_6143_MOESM3_ESM.docx]

**Additional file 3: Table S1** The sequence of primers related to PCR amplication

| Gene name | Forward primer(5’-3’) | Reverse primer(5’-3’) |
| --- | --- | --- |
| *SbER1-1* DNA | TCTCTCTCATCAGTCATCACAAGAAACCGC | TCAAGAAAGTCACCTCCCATGATTCCAACACG |
| *SbER2-1* DNA | GTGAAGTGAGGACCAATGCGGCAGTGAACCACCCCAAGCTTTCTAGAGGATCCATGCCTGTC | TGCCGGTGCTAACCCAAGCGACAGTGCAGTG |
| *SbER1-1* cDNA | CGCAGCTCAGTGGCCATGACGACGACGGCCGCCCGTGCTCT | CGGGGTACCCTACTCCATGTTCTGCGAGATG |
| *SbER2-1* cDNA | AAGGCCTATGGCCCGCCTCCTCCGGGCCCTCGCCGCCCTCCTCC | CGGGGTACCACTAGTCTACTCCGTGCTTCGCGATATCACCTCG |
| *Ubi*-1 | CCCAAGCTTGCATGCCTGCAGTG | CGGGATCCTCTAGAGTCGACCTGCAGAA |
| *Ubi*-2 | CCCAAGCTTGCATGCCTGCAGTG | AAGGCCTTCTAGAGTCGACCTGCAGAA |
| *SbER2-1*(VK011) | CTTTGAAAAAGAGGGGGATTAATGGCCCGCCTCCTCCGGGCCCTCG | TGATTTCAGCGTACCGAATTGTTACCTACTCCGTGCTTCGCGATATCAC |

Note: Long fragment specific amplification primers were designed according to the sequence of *SbER1* and *SbER2* in NCBI database information, and the restriction sites (underline) for cloning ligation were added.
