# Supplementary material for: A leucine-rich repeat-receptor-like kinase gene SbER2–1 from sorghum (Sorghum bicolor L.) confers drought tolerance in maize
Source: BMC Genomics. 2019 Oct 15;20:737. doi: 10.1186/s12864-019-6143-x (PMC6794760; doi:10.1186/s12864-019-6143-x)
Supplement: Supplementary file 4 — Additional file 4: Table S2. The qRT-PCR primers of SbER1–1, SbER2–1 and alpha-tubulin [file 12864_2019_6143_MOESM4_ESM.docx]

**Additional file 4: Table S2** The qRT-PCR primers of *SbER1-1*, *SbER2*-1 and *alpha-tubulin*

| name | | Primer（5'-3'） | length  (mer) | Tm  (℃) | GC% | product（bp） |
| --- | --- | --- | --- | --- | --- | --- |
| *SbER1-1* | 115GL1-F | GGCTATGGCATTGTTCTGCTG | 21 | 59.93 | 52.38 | 131 |
|  | 115GL1-R | CCGATGTCAGGGTCCACG | 18 | 59.82 | 66.67 |  |
| *SbER2-1* | 115GL2-F | TGCAATCTTCATCACTTGATCC | 22 | 56.76 | 40.91 | 108 |
|  | 115GL2-R | CTTCTTGACCTCACCAAGGTCT | 22 | 59.63 | 50.00 |  |
| *alpha-*  *tubulin* | Ptu-F | GTGCATCTCGATCCACATTG | 20 | 56.97 | 50.00 | 145 |
|  | Ptu-R | GGTGTTGAAAGCATCGTCAC | 20 | 57.68 | 50.00 |  |
